# Clinicopathologic Classification of Focal Segmental Glomerulosclerosis to Inform on Outcomes: A Retrospective Cohort Review of Biopsy-Proven Focal Segmental Glomerulosclerosis Cases

**DOI:** 10.1016/j.xkme.2026.101364

**Published:** 2026-04-14

**Authors:** Jennifer Horwitz, Ayub Akbari, Lina Rahouma, Mark Canney, David Massicotte-Azarniouch

**Affiliations:** 1 Department of Medicine, University of Ottawa, Ottawa, Ontario, Canada; 2 Division of Nephrology, Department of Medicine, University of Ottawa, Ontario, Canada; 3 Clinical Epidemiology Program, Ottawa Hospital Research Institute, Ottawa, Ontario, Canada

**Keywords:** Focal segmental glomerulosclerosis (FSGS), glomerulonephritis, immunosuppression, podocytopathy

To the Editor:

Focal segmental glomerulosclerosis (FSGS) is a histologic pattern of glomerular injury. Historically classified by histologic variant, FSGS is now commonly categorized as primary (idiopathic or immune-mediated), secondary (adaptive, medication-induced, or viral-associated), or genetic.^1^^,^^2^ Despite shared podocyte injury, outcomes vary widely, with up to 50% progressing to kidney failure.^3^, ^4^, ^5^, ^6^ Accurate subclassification is essential for prognostication and treatment decisions, particularly regarding immunosuppression (IS), but it remains challenging due to limited access to genetic testing and lack of definitive biomarkers for primary FSGS.^7^^,^^8^

A previous study^9^ showed that classification of FSGS using clinicopathologic features (serum albumin, degree of proteinuria, and podocyte foot process effacement [FPE]) can enhance patient selection for genetic testing. Our study evaluated whether this classification is associated with clinical outcomes.

We conducted a retrospective cohort study of patients with biopsy-proven FSGS at The Ottawa Hospital (2010-2023). Patients were classified as presumed primary FSGS (serum albumin <35 g/L, proteinuria >3.5 g/d, and diffuse FPE >80%), presumed secondary FSGS (serum albumin ≥35 g/L, no diffuse FPE, regardless of proteinuria level), or uncategorized FSGS (not meeting either definition). Outcomes were complete and partial proteinuria remission (CR and PR), kidney failure, death, and change in estimated glomerular filtration rate (eGFR). Associations of FSGS classification to outcomes were assessed using Cox regression adjusted for age, sex, baseline eGFR, and interstitial fibrosis/tubular atrophy. Full methods are available in the Supplementary Material (Item S1).

After exclusions, 187 patients were analyzed (Figure S1, Table S1). Mean age (± standard deviation) was 53.8 ± 15.6 years; 59.4% were male. Mean creatinine at biopsy was highest in presumed primary FSGS (209 ± 179.3 μmol/L). As expected by diagnostic assignment, this group also had the lowest serum albumin (22.8 ± 7.7 g/L) and highest proteinuria (9.2 ± 7.3g/d). At baseline, 55.6% of patients used renin–angiotensin system inhibitors and 4.8% sodium/glucose cotransporter 2 inhibitors.

Main outcomes are summarized in Table 1, and as further stratified by histologic classification (Table S2) and treatment received (Table S3). Over a mean follow-up of 3.8 ± 3.1 years, 17.6% achieved CR (29.6% of presumed primary, 15.3% of secondary, 9.8% of uncategorized). Presumed primary FSGS had a higher likelihood of CR than presumed secondary (adjusted hazard ratio [HR], 2.30; 95% confidence interval [CI], 1.01-5.22). PR occurred in 40.7%, 58.3%, and 60.7% of presumed primary, secondary, and uncategorized groups, respectively, with presumed primary FSGS being significantly less likely to achieve PR than presumed secondary (adjusted HR, 0.53; 95% CI, 0.31-0.90). Kidney failure occurred in 20.9% of patients, with mean time to kidney failure shortest in presumed primary FSGS (1.4 vs 3.9 and 2.6 years), although differences were not statistically significant (adjusted HR, 0.59; 95% CI, 0.24-1.47).

**Table 1:** Outcomes, by Category of FSGS

	Full Cohort	Presumed Primary FSGS	Presumed Secondary FSGS	Uncategorized
Number of patients, n (%)	187	54 (28.9%)	72 (38.5%)	61 (32.6%)
Follow-up, y, mean (SD)	3.8 (3.1)	3.9 (3.2)	3.6 (3.0)	4.0 (3.1)
**Remission of proteinuria**				
Complete^a^, n (%)	33 (17.6%)	16 (29.6%)	11 (15.3%)	6 (9.8%)
HR (95% CI)	—	2.22 (1.03, 4.79)	Ref	0.61 (0.23, 1.64)
aHR (95% CI)	—	2.30 (1.01, 5.22)	Ref	0.64 (0.23, 1.75)
Partial^b^, n (%)	101 (54.0%)	22 (40.7%)	42 (58.3%)	37 (60.7%)
HR (95% CI)	—	0.54 (0.32, 0.91)	Ref	0.94 (0.61, 1.47)
aHR^c^ (95% CI)	—	0.53 (0.31, 0.90)	Ref	0.91 (0.58, 1.43)
**Kidney failure**				
Kidney failure, n (%)	39 (20.9%)	10 (18.5%)	12 (16.7%)	17 (27.9%)
Time to event y, mean (SD)	2.7 (2.6)	1.4 (2.2)	3.9 (3.1)	2.6 (2.2)
HR (95% CI)	—	1.05 (0.45, 2.42)	Ref	1.54 (0.73, 3.22)
aHR^c^ (95% CI)	—	0.59 (0.24, 1.47)	Ref	0.98 (0.43, 2.22)
**Death**				
Death, n (%)	23 (12.3%)	6 (11.1%)	8 (11.1%)	9 (14.8%)

Abbreviations: aHR, adjusted hazard ratio; CI, confidence interval; FSGS, focal segmental glomerulosclerosis; HR, hazard ratio; Ref, reference.

a Defined as proteinuria <0.3 g/d.

b Defined as proteinuria <3.5 g/d and >50% reduction from baseline, without achieving complete remission.

c HR adjusted for age, sex, kidney function at biopsy and degree of interstitial fibrosis/tubular atrophy on biopsy.

IS within 6 months of biopsy was used in 42.6% of presumed primary, 1.4% of secondary, and 16.4% of uncategorized FSGS (Table S4). Among presumed primary FSGS, IS-treated patients had higher CR rates (42.9% vs 21.2%). IS-treated presumed primary FSGS also showed early post-biopsy eGFR improvement, which was not seen in other groups (Figure S2, Table S7 and eGFR in presumed primary FSGS remained significantly higher than presumed secondary FSGS throughout (Fig 1, Table S5); the uncategorized group followed a trajectory similar to that of secondary FSGS (Figure S3, Table S6).

**Figure 1:**
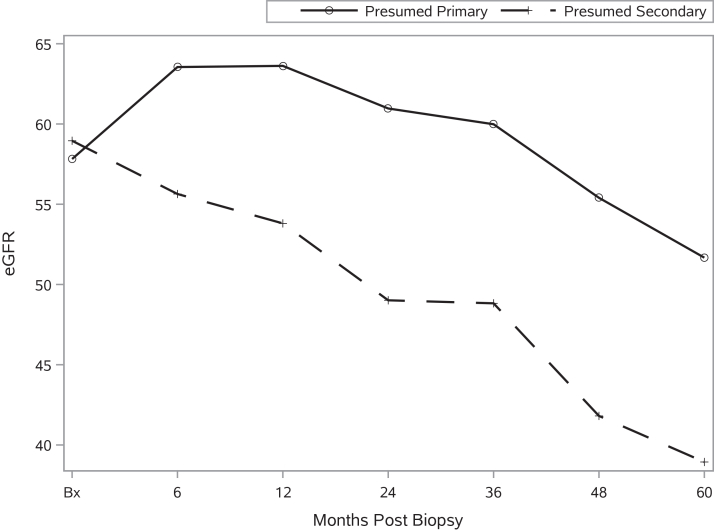
Estimated glomerular filtration rate (eGFR) over time for presumed primary and presumed secondary focal segmental glomerulosclerosis.

These findings suggest that classification using albumin, proteinuria, and FPE may identify FSGS subgroups with distinct clinical courses. Unlike prior studies that combine PR and CR, we evaluated these outcomes separately, revealing important differences. Patients with presumed primary FSGS were more likely to achieve CR, respond to IS, and show eGFR improvement, but less likely to achieve PR, which, by definition, excluded CR. This pattern may suggest an ‘all-or-nothing’ response in presumed primary (likely immune-mediated) FSGS, where patients who respond to early IS tend to achieve full remission, whereas nonresponders may remain resistant to therapy. This may also account for the shorter time to kidney failure in presumed primary FSGS, driven by rapid progression among nonresponders. In contrast, higher PR in secondary and uncategorized groups likely reflects responses to supportive, antiproteinuric therapies. The early eGFR improvement in primary FSGS, particularly among IS-treated patients, may suggest a true treatment effect and recovery from proteinuria-related injury. Notably, repeating our analyses using contemporary FSGS trial definitions of CR and PR yielded findings generally consistent with the primary analysis (Table S8).^10^

Limitations include the retrospective design, single-center setting, variable follow-up (mean 3.8 years) that was insufficient for long-term outcomes, and limited genetic testing (Table S1). Categorization using albumin, proteinuria, and FPE is arbitrary and somewhat subjective, with unavoidable overlap between groups, leading to potential misclassification. Accordingly, some uncategorized patients, particularly those with nephrotic syndrome, may still benefit from IS. Nonetheless, this represents one of the larger biopsy-proven FSGS cohorts evaluated using this framework.

Our findings support the value of a clinicopathologic classification system for FSGS. Although secondary causes or advanced kidney dysfunction may dissuade from the use of IS, clinicopathologic indicators of primary FSGS using serum albumin, proteinuria, and degree of FPE may identify patients most likely to benefit.

## Supplementary Materials

Supplementary File (PDF) Item S1; Table S1-S8; Figure S1-S3
