# Supplementary material for: Clinicopathologic Classification of Focal Segmental Glomerulosclerosis to Inform on Outcomes: A Retrospective Cohort Review of Biopsy-Proven Focal Segmental Glomerulosclerosis Cases
Source: Kidney Med. 2026 Apr 14;8(6):101364. doi: 10.1016/j.xkme.2026.101364 (PMC13196506; doi:10.1016/j.xkme.2026.101364)

Supplementary Materials

Item S1. Full Methods

Study design and setting

We performed a retrospective, single-centre cohort study of all patients diagnosed at The Ottawa Hospital (TOH) with FSGS on native kidney biopsy. The study period was from 1 January 2010 until 30 December 2023 (data collected until 30 August 2024). TOH is a 1200-bed, 3-campus academic teaching hospital, and is the largest tertiary care referral center for adults in a region of more than 1.2 million residents. The TOH Glomerulonephritis Clinic Renal Pathology Database was used to identify the study population and baseline characteristics; this database is regularly maintained by a trained clerk and contains all native kidney biopsies performed at TOH, with histopathologic diagnosis, pathology variables and clinical variables from time of biopsy. The electronic patient chart was reviewed by the study team to confirm the diagnosis of FSGS, ascertain exposures and outcomes. One participant was excluded because they did not have FSGS on review of the chart. Participants without mention of degree of FPE on EM in the biopsy report were also excluded since this information was required for exposure classification. Participants without data on serum albumin at time of biopsy were likewise excluded. There were otherwise no exclusions. Institutional research and ethics board approval was received before data gathering and study analysis. Due to the retrospective nature of our study and use of de-identified data, informed consent was waived. The study design, exposure, outcomes, and analysis were all determined before data retrieval and analysis.

Exposure categories

Study participants were classified into 3 categories of FSGS: 1) Presumed primary FSGS (at time of biopsy, serum albumin <35g/L AND proteinuria >3.5g/day AND diffuse FPE [>80%] on electron microscopy); 2) Presumed secondary FSGS (at time of biopsy, serum albumin ≥35g/L AND no diffuse FPE on electron microscopy, regardless of level of proteinuria); 3) Uncategorized FSGS (does not fit into one of the 2 categories above).

Outcomes

All outcomes were captured through chart review: complete remission (daily urinary protein excretion <0.3g/day), partial remission (proteinuria <3.5g/day and >50% reduction of from baseline, without having achieved complete remission), kidney failure (requiring at least 12 weeks of regular dialysis or initiation of evaluation for a kidney transplant), death, and change in estimated glomerular filtration rate (eGFR). Lab values were collected at 6 months, 12 months, 18 months, 24 months, and then yearly, from time of biopsy. Receipt of immunosuppression for the treatment of FSGS (within the first 6 months after biopsy) and clinical lab values during follow-up were also captured. Serum creatinine (umol/L) was used to calculate eGFR (ml/min/1.73m^2) using the CKD-EPI 2021 non race-based formula. Daily urinary protein excretion was estimated using urine protein-creatinine ratio (Upcr, g/g) by converting urine albumin-creatinine ratios (mg/mmol) to Upcr (mg/mmol) then dividing by 113 to get Upcr in g/g.[S1]

Analysis

Baseline characteristics, exposures and outcomes are presented for the total cohort and by exposure category. The start of follow-up was the day of kidney biopsy. Descriptive statistics, means with standard deviations (SD), medians with interquartile ranges (IQR) and counts with percentages, were presented with the appropriate test statistic. Cox proportional hazards regression was used to examine the association between FSGS category and time-to-event outcomes, using the Presumed Secondary FSGS category as the reference and adjusted for baseline age, sex, eGFR, and interstitial fibrosis and tubular atrophy (IFTA). Outcomes were also examined for FSGS categories, by receipt of immunosuppression or not. Generalized linear mixed models, adjusted for baseline age, sex, eGFR, and IFTA, were used to examine change in mean eGFR throughout follow-up, by FSGS category. Serum creatinine measurements were analyzed up to the development of kidney failure or end of follow-up, whichever occurred first. Values obtained after kidney failure onset were censored, and no imputation was performed for missing data. All analyses were performed with SAS software (v9.4, SAS Institute).

Supplementary References

S1. Deegens JKJ, Steenbergen EJ, Meijer E, van den Berg JG, van der Vlag J, Jennette JC, et al. Idiopathic focal segmental glomerulosclerosis: a distinct entity with heterogeneous histologic and clinical features. *Kidney Int*. 2008 Sep;74(5):548–56.

S2. D'Agati VD, Fogo AB, Bruijn JA, Jennette JC. Pathologic classification of focal segmental glomerulosclerosis: a working proposal. Am J Kidney Dis. 2004 Feb;43(2):368–82.

Supplementary Tables and Figures

Table S1. Baseline characteristics of study population, by category of FSGS

	**Total, N = 187**	**Presumed primary FSGS**	**Presumed secondary FSGS**	**Uncategorized**
Number of patients, N	187	54	72	61
Age, years; mean (SD)	53.8 (15.6)	53.3 (16.7)	54.3 (15.1)	53.5 (15.5)
Gender				
Male, N (%)	111 (59.4)	28 (51.9)	48 (66.7)	35 (57.4)
BMI, kg/m^2, mean (SD)	30.4 (7.1)	29.3 (7.9)	30.6 (6.1)	31.2 (7.6)
Comorbidities at time of biopsy				
Diabetes[*], N (%)	34 (18.3)	8 (14.8)	16 (22.2)	10 (16.7)
Hypertension[#], N (%)	132 (71.4)	32 (60.4)	53 (73.6)	47 (78.3)
Dyslipidemia[+], N (%)	90 (48.1)	22 (40.7)	37 (51.4)	31 (50.8)
Smoking status at time of biopsy				
Active smoker, N (%)	46 (25.0)	14 (25.9)	14 (20.0)	24 (40.0)
Positive family history of kidney disease, N (%)	12 (6.4)	4 (7.4)	5 (6.9)	3 (4.9)
Known cause of secondary FSGS at time of biopsy[Ω], N (%)	46 (24.6)	7 (13.0)	27 (37.5)	12 (19.7)
Genetic testing completed, N (%)	10 (5.35)	4 (7.4)	4 (5.6)	2 (3.3)
Genetic testing confirmed genetic cause of FSGS, N (%)**	4 (40.0)	1 (25.0)	0 (0)	2 (100.0)
Medication use at time of biopsy				
ACEi/ARB, N (%)	104 (55.6)	23 (42.6%)	47 (65.3)	34 (55.7)
SGLT2i, N (%)	9 (4.8)	1 (1.9)	5 (6.9)	3 (4.9)
**Biopsy characteristics**				
Histological classification[S2]				
Collapsing, N (%)	6 (3.2)	5 (9.4)	0	1 (1.6)
Peri-hilar, N (%)	28 (15.1)	5 (9.4)	12 (16.7)	11 (18.0)
Tip, N (%)	18 (9.7)	15 (28.3)	0	3 (4.9)
Cellular, N (%)	4 (2.2)	3 (5.7)	1 (1.4)	0
NOS, N (%)	130 (69.9)	25 (47.2)	59 (81.9)	46 (75.4)
IFTA				
None/minimal, N (%)	18 (9.6)	11 (20.4)	4 (5.6)	3 (4.9)
Mild, N (%)	83 (44.4)	18 (33.3)	39 (54.2)	26 (42.6)
Moderate, N (%)	53 (28.3)	16 (29.6)	16 (22.2)	21 (34.4)
Severe, N (%)	33 (17.7)	9 (16.7)	13 (18.1)	11 (18.0)
**Clinical characteristics at biopsy**				
Serum creatinine in μmol/L, mean (SD)	167.9 (12.4)	209.0 (179.3)	141.2 (65.7)	163.0 (108.6)
Serum albumin in g/L, mean (SD)	33.4 (9.2)	22.8 (7.7)	40.8 (3.5)	33.9 (5.2)
Daily protein excretion in grams, mean (SD)	4.49 (5.2)	9.2 (7.3)	2.0 (1.2)	3.2 (2.4)

[*]Known diagnosis of diabetes or HbA1c >/= 6.5% or random PG > 11.1 mmol/L

#Known diagnosis of hypertension or on anti-hypertensives at time of biopsy
+Known diagnosis of dyslipidemia or LDL-c >/= 3.5 mmol/L or non-HDL-c >/= 4.3 mmol/L or on Statin medication at time of biopsy
ΩObesity (BMI > 30 mg/kg^2 at time of biopsy), history of prior nephrectomy or partial nephrectomy, obstructive sleep apnea, reflux nephropathy, history of congenital anomalies of kidney or urinary tract or premature birth, viral infection (HIV, Parvovirus B19 or COVID-19), exposure to medication associated with FSGS (bisphosphonates, lithium, interferon, calcineurin inhibitors, anabolic steroids), history of prior renal insult
**Genetic variants identified: PAX2 gene variant (in one patient), Autosomal Dominant Alport's syndrome (in 3 patients)

Table S2. Outcomes, by histologic classification of FSGS

	Collapsing (N=6)	Peri-hilar (N=28)	Tip (N=18)	Cellular (N=4)	NOS (N=130)
CR; N (%)	1 (16.7)	3 (10.7)	10 (55.6)	1 (25.0)	17 (13.1)
CR or PR; N (%)	4 (66.7)	23 (82.1)	15 (83.3)	3 (75.0)	88 (47.3)
Kidney failure; N (%)	2 (33.3)	6 (21.4)	0	0	31 (23.9)

Table S3. Outcomes, further stratified by treatment received

	**Presumed primary FSGS (54)**		**Presumed secondary FSGS (72)**		**Uncategorized (61)**	
	**IS YES**	**IS NO**	**IS YES**	**IS NO**	**IS YES**	**IS NO**
Number of patients, N (%)	21 (42.6)	33 (57.4)	1 (1.4)	71 (98.6)	10 (16.4)	51 (83.6)
Remission of proteinuria<br>Complete[β], N (%)<br>Partial[θ], N (%)	 9 (42.9)<br>10 (47.6)	 7 (21.2)<br>12 (36.4)	 0<br>0	 11 (15.5)<br>42 (59.2)	 1 (10.0)<br>6 (60.0)	 5 (9.8)<br>31 (60.8)

βDefined as proteinuria < 0.3 g/day
θDefined as proteinuria <3.5g/day and > 50% reduction from baseline, without achieving complete remission

Table S4. Frequency of use of immunosuppression (IS) in the first 6 months, by category of FSGS

	**Presumed primary FSGS (54)**	**Presumed secondary FSGS (72)**	**Uncategorized (61)**
No IS use, N (%)	31 (57.4)	71 (98.6)	51 (83.6)
Prednisone use, N (%)	21 (38.9)	1 (1.4)	8 (13.1)
Calcineurin inhibitor use, N (%)	1 (1.9)	0	1 (1.6)
Mycophenolate use, N (%)	1 (1.9)	0	1 (1.6)
Cyclophosphamide use, N (%)	0	0	0
Rituximab use, N (%)	0	0	0

Table S5. Change in eGFR between presumed primary FSGS and presumed secondary FSGS, adjusted for age, sex, eGFR, and IFTA at biopsy

Time point	Presumed primary FSGS	Presumed secondary FSGS	ΔGFR primary minus ΔGFR secondary (*p-value*)
Biopsy	57.8 (53.8, 61.8)	59.0 (55.5, 62.4)	-
6 months	63.5 (59.3, 67.8)	55.6 (52.1, 59.2)	7.9 (0.005)
12 months	63.2 (58.9, 68.3)	53.8 (49.8, 57.8)	9.8 (0.002)
24 months	61.0 (55.9, 66.0)	49.0 (44.5, 53.5)	12.0 (<0.001)
36 months	60.0 (54.9, 65.1)	48.8 (44.0, 53.6)	11.2 (0.002)
48 months	55.4 (50.0, 60.8)	41.8 (36.5,47.2)	13.6 (<0.001)
60 months	51.7 (45.7, 57.7)	38.9 (33.3, 44.6)	12.7 (0.003)

Table S6. Change in eGFR between presumed secondary FSGS and uncategorized FSGS, adjusted for age, sex, eGFR, and IFTA at biopsy

Time point	Uncategorized FSGS	Presumed secondary FSGS	ΔGFR uncategorized minus ΔGFR secondary (p-value)
Biopsy	59.5 (56.5, 62.5)	59.6 (56.8, 62.3)	-
6 months	56.2 (53.1, 59.3)	56.3 (53.5, 59.1)	-0.1(0.96)
12 months	57.1 (53.6, 60.5)	54.5 (51.3, 57.6)	2.6 (0.28)
24 months	54.7 (51.0, 58.3)	49.4 (45.9, 52.9)	5.3 (0.04)
36 months	49.3 (45.4, 53.2)	49.0 (45.2, 52.8)	0.3 (0.92)
48 months	46.5 (42.3, 50.6)	41.8 (37.7, 46.0)	4.6 (0.13)
60 months	44.9 (40.6, 49.1)	39.1 (34.8, 43.5)	5.7 (0.07)

Table S7. Change in eGFR among presumed primary FSGS, by receipt of immunosuppression, adjusted for age, sex, eGFR and IFTA at biopsy

Time point	Treated with IS	No IS	ΔGFR IS minus ΔGFR no IS	p-value (IS vs no IS)
Biopsy	52.6 (44.5, 60.6)	55.8 (49.3, 62.2)	-	-
6 months	65.2 (56.9, 73.5)	56.8 (49.8, 63.7)	8.4	0.13
12 months	62.4 (54.2, 70.6)	59.5 (51.1, 67.9)	2.9	0.63
24 months	67.0 (57.7, 76.4)	51.0 (42.5, 59.4)	16.1	0.01
36 months	62.7 (53.5, 71.9)	52.2 (43.5, 61.0)	10.5	0.11
48 months	58.4 (48.4, 68.4)	47.5 (38.3, 56.6)	10.9	0.12
60 months	53.8 (43.2, 64.4)	44.4 (33.8, 55.0)	9.4	0.22

Table S8. Outcomes, by category of FSGS, using contemporary definitions of CR and PR

*Partial remission in this analysis was defined as: attained a urine protein to creatinine ratio <

	Full Cohort	Presumed primary FSGS	Presumed secondary FSGS	Uncategorized FSGS
Number of patients, N (%)	187	54 (28.9)	72 (38.5)	61 (32.6)
**Complete remission of proteinuria*, N (%)**	64 (34.2)	21 (38.9)	26 (36.1)	17 (27.9)
HR (95% CI)		1.201 (0.675-2.136)	Ref	0.769 (0.417 – 1.420)
aHR (95% CI)		1.201 (0.654 – 2.239)	Ref	0.824 (0.444 – 1.529)
**Partial remission of proteinuria**, N (%)**	41 (21.9)	7 (13.0)	20 (27.8)	14 (23.0)
HR (95% CI)		0.420 (0.177 – 0.993)	Ref	0.717 (0.362 – 1.421)
aHR (95% CI)		0.382 (0.155 – 0.943)	Ref	0.671 (0.334 – 1.349)
**Composite outcome (eGFR decrease by 40%, kidney failure or death), N (%)**	55 (70.6)	15 (27.8)	17 (23.6)	23 (37.7)
HR (95% CI)		1.110 (0.553 – 2.226)	Ref	1.467 (0.783 – 2.749)
aHR (95% CI)		0.623 (0.292 – 1.331)	Ref	1.164 (0.596 – 2.274)

170 mg/mmol and > 40% decrease in urine protein-to-creatinine ratio from baseline AND did not achieve complete remission

**Complete remission in this analysis was defined as: attained a urine protein-to-creatinine ratio < 79 mg/mmol.

Figure S1. Study cohort selection

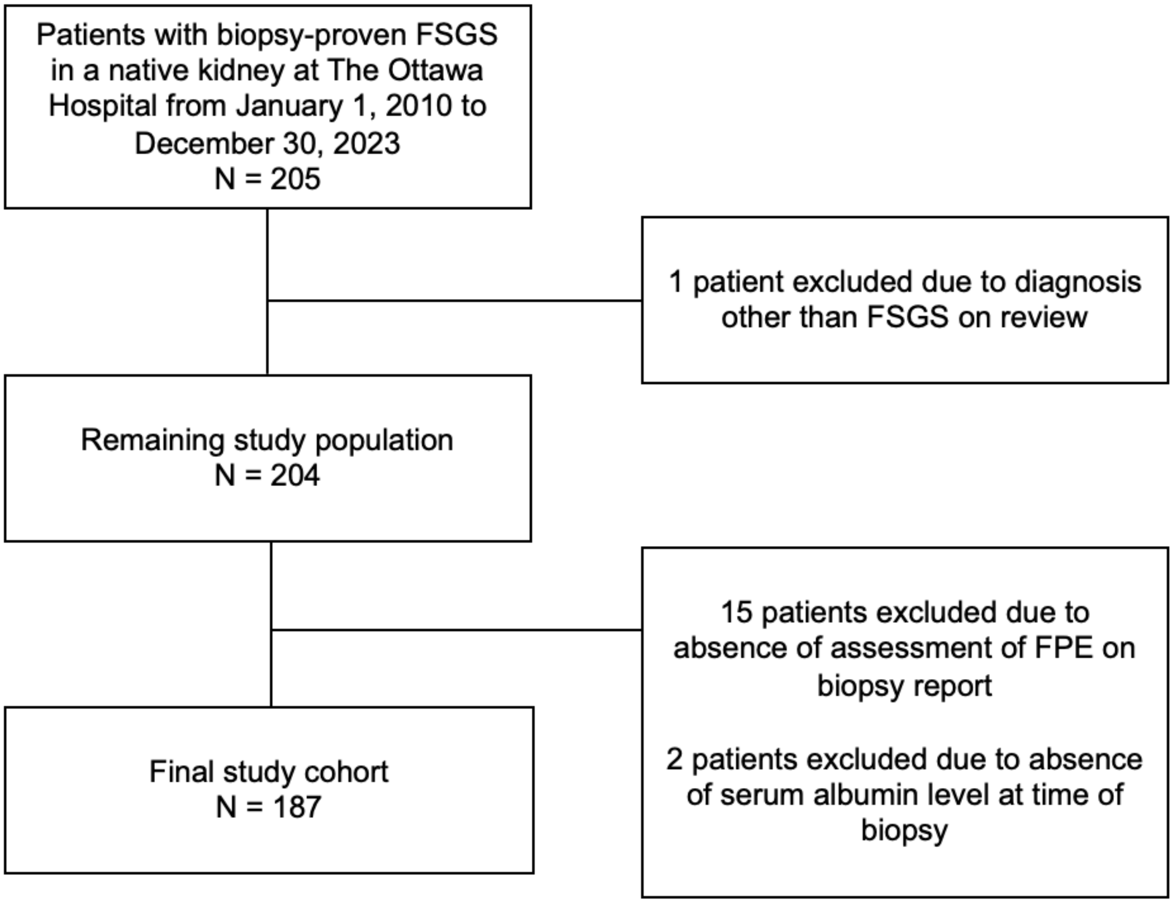

Figure S2. eGFR over time for presumed primary FSGS, stratified by receipt of immunosuppression

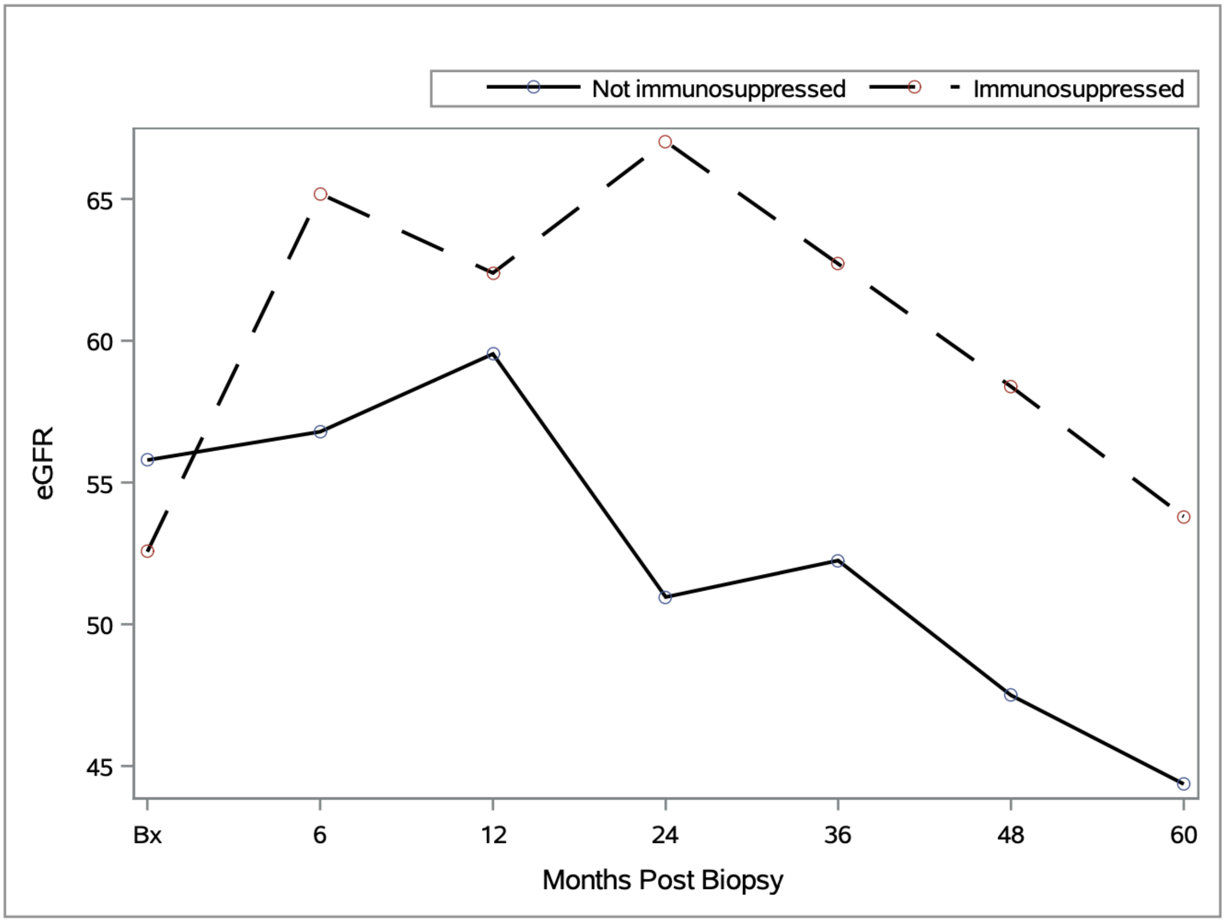

Figure S3. Linear mixed model of mean eGFR, adjusted for baseline age, sex, eGFR and IFTA for presumed secondary and uncategorized FSGS

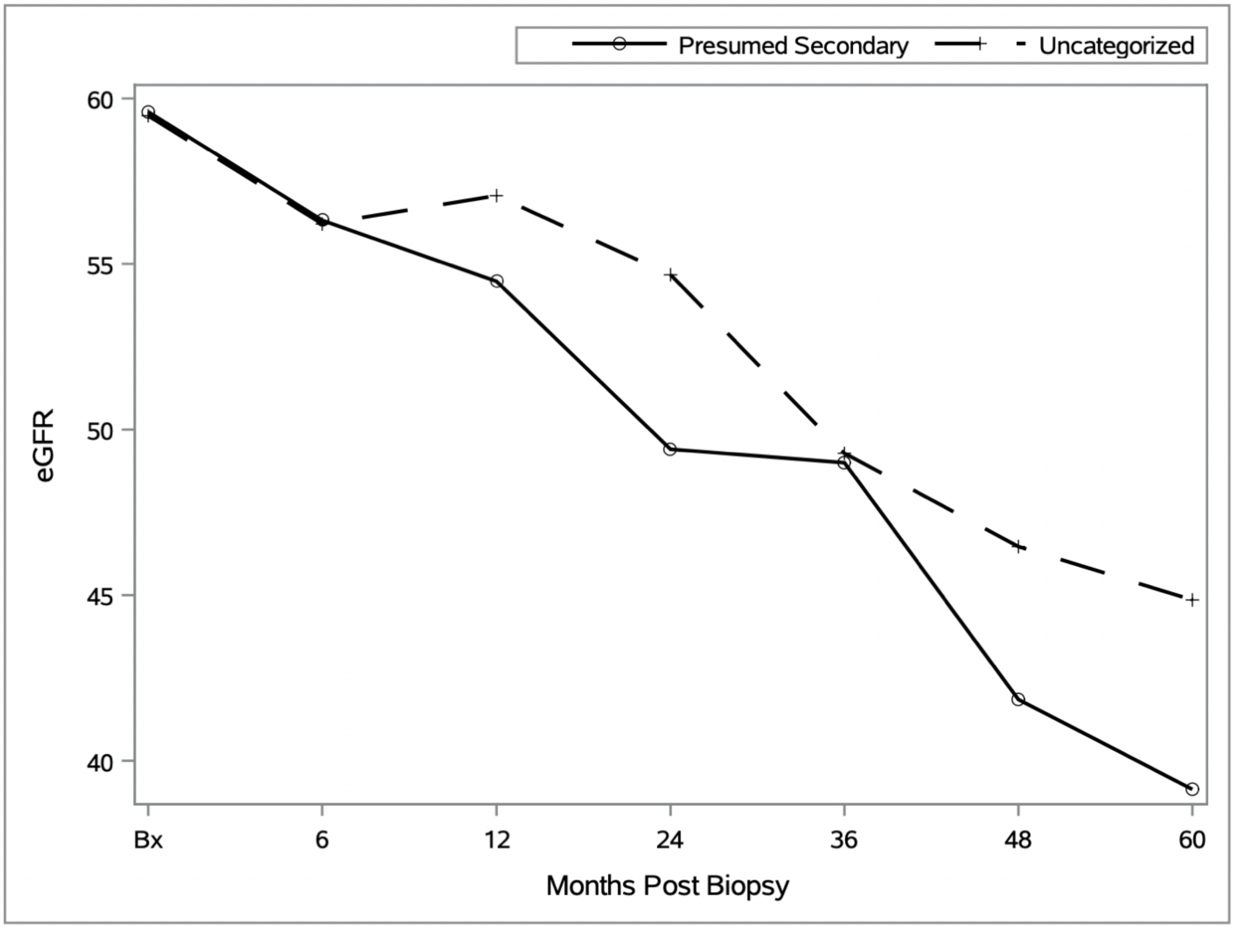

Supplement: Supplementary File (PDF) — Item S1; Table S1-S8; Figure S1-S3 [file mmc1.pdf]
